# One-Year Post Hoc Analysis of Renal Function for Live Kidney Donors That Were Enrolled in an Enhanced Recovery After Surgery Pathway With Ketorolac

**DOI:** 10.7759/cureus.10056

**Published:** 2020-08-26

**Authors:** Jeffrey Campsen, Chong Zhang, Angela Presson, Mariah Goodale, Gilbert Pan, Sarah Scutts, George Rofaiel, Robin Kim

**Affiliations:** 1 Department of Surgery, Division of Transplantation and Advanced Hepatobiliary Surgery, University of Utah School of Medicine/Huntsman Cancer Institute, Salt Lake City, USA; 2 Department of Medicine, Division of Epidemiology, University of Utah School of Medicine/Huntsman Cancer Institute, Salt Lake City, USA

**Keywords:** live donor, nephrectomy, kidney transplant, narcotic

## Abstract

Background and Objective

Opioid exposure is a concern after live donation for kidney transplants (LDKT). We previously theorized that an enhanced recovery after surgery (ERAS) pathway for LDKT will reduce perioperative narcotic use. The aim of this post hoc analysis of merged data from two ERAS trials was to review the one-year follow-up to determine if the exposure to ketorolac versus placebo had any significant impact on long-term kidney function after LDKT.

Methods

One-year post hoc analysis of merged data from two ERAS LDKT, prospective, double-blind, randomized clinical trials were combined involving a total of 72 patients undergoing nephrectomy for LDKT. Kidney functions of both the ERAS groups' versus placebo were compared prospectively and blinded at one year using estimated glomerular filtration rate (eGFR) and total protein (TP) in the urine in compliance with United Network for Organ Sharing (UNOS) live donor requirements.

Results

There was no significant difference in postoperative eGFR at one year between ERAS and placebo groups. TP urine at one-year post-operative was significantly lower in the ERAS cohort by 4.7 mg/dl (95% CI 0.48 ~ 8.82, p = 0.025).

Conclusions

The ERAS groups' exposure to ketorolac did not negatively affect kidney function at one year after LDKT.

## Introduction

Live donors for kidney transplants risk opioid exposure following the surgery, which is a concern given the current opioid crisis. Narcotic alternatives such as ketorolac have reported risk for acute renal failure when used for surgical procedures in the literature [1]. Few retrospective studies in transplant literature can be found that address narcotic alternatives [2,3], and even fewer long-term data exist. This is a long-term post hoc analysis of merged data from two ERAS trials at a single center [4,5] seeking to understand how an enhanced recovery after surgery (ERAS) pathway using non-narcotic pain management including ketorolac versus standard of care (SOC) plus placebo in live donor nephrectomy transplant surgery affects kidney function.

Ketorolac is a nonsteroidal anti-inflammatory drug (NSAID) that acts by inhibiting the synthesis of prostaglandins. The competitive inhibition of the enzyme cyclooxygenase (COX) mediates the anti-inflammatory and analgesic effects derived from ketorolac. ERAS studies using ketorolac in live donors for kidney transplantation have shown positive results [6], including our own center's two studies [4,5]; but the long-term effects on the donor's remaining kidney have not been evaluated. We theorized that an ERAS protocol utilizing ketorolac after surgery is safe for the donor's remaining kidney long term based on our perioperative study results. This study aims to review one-year follow-up of two studies still blinded and randomized to determine if the exposure to ketorolac versus placebo had any significant impact on long-term kidney function after live donor nephrectomy.

## Materials and methods

One-year post hoc analysis of merged data from two single center, prospective, double-blind, randomized clinical trials were combined involving a total of 72 patients undergoing laparoscopic hand-assisted nephrectomy (no patients were converted to open) for live donor kidney transplantation during 2015-2017 [4,5]. Kidney functions of both the ERAS pathway including the NSAID and ketorolac versus placebo were compared prospective, double-blind, and randomized. At one year, kidney function was evaluated using estimated glomerular filtration rate (eGFR) and total protein (TP) in the urine in compliance with United Network for Organ Sharing (UNOS) live donor requirements [7]. The chronic kidney disease epidemiology collaboration (CKD-EPI) equation is one of the most widely used equations to estimate glomerular filtration rate (GFR) from serum creatinine [8]. The equation utilizes age, race, and sex as variables associated with muscle mass for serum creatinine in assessing GFR [9].

This study was approved by the University of Utah Institutional Review Board. After informed consent, patients were randomized to one of two arms by the University of Utah lnvestigational Drug Pharmacy. The criteria for inclusion were age greater than 18 and should be undergoing an elective donor nephrectomy for renal donation. Patients were excluded for any of the following causes: substance abuse, history of chronic pain, nephrectomy for reasons other than donation, preoperative elevated creatinine above 1.5 mg/dL, and pregnant or lactating females.

Patients were randomized in a double-blind fashion to either Arm 1 (ERAS study group) or Arm 2 (placebo). The first study was a pilot trial of 15 patients, and the second trial included gabapentin. Investigational drug pharmacists collaborating on this study were not blinded and prepared the corresponding study drugs through a web-based format indicating assignment based on each subject's ascertainment order. In addition to the randomized arm assignment, patients were given all standard of care pain medications (no patient-controlled analgesia) without any withholding of narcotics as deemed necessary by the patient. Standard of care included intraoperative narcotics that were controlled and postoperative narcotic availability. All study patients had IV narcotics available and were switched to oral narcotics after tolerating liquids by mouth. Patients were followed throughout the hospital stay and discharged to home. The use of ketorolac was consistent with the US Food and Drug Administration approval in terms of route of administration and dosing; therefore, this is exempt from investigational new drug requirement.

Patient demographics and clinical measures were summarized descriptively and compared between the ERAS and placebo group. Continuous variables were summarized as mean (standard deviation: SD), median (interquartile range: IQR), and range and compared using t-tests. Categorical variables were summarized as frequency (%) and compared using chi-squared or Fisher's exact tests. TP urine was left censored at 7 mg/dl due to the detection limit of the laboratory and was compared between groups using tobit regression, treating values below 7 mg/dl as 6.9 mg/dL [10,11]. Tobit regression is a regression technique that is appropriate for outcome variables with left or right censoring. Tobit regression coefficients can be interpreted similarly to conventional linear regression analysis, although their effect corresponds to an uncensored latent version of the TP urine outcome variable [12].

Our primary outcome, postoperative eGFR, was compared between treatment groups using linear regression adjusting for preoperative eGFR level. Adjustment for preoperative eGFR provides the structure of an analysis of covariance (ANCOVA), which has greater statistical power than alternative approaches [13,14]. Our secondary outcome, postoperative TP urine, was compared using tobit regression as described previously. We were unable to adjust for preoperative TP urine in this comparison because our preoperative TP level was processed by a separate laboratory, and results were not comparable between laboratories due to differing processing protocols. For both outcomes, regression coefficients were reported with 95% confidence intervals (CIs) and p values. Statistical significance was assessed at the 0.05 level, and all tests were two-tailed. All analyses were conducted using R 3.5.1.

## Results

There were 38 patients in the ERAS cohort and 34 in placebo. Demographics and endpoints are reported in Table 1. Women represented 78.9% of the ERAS cohort and 52.9% of the placebo cohort (p* *= 0.019), but there were no statistically significant differences in other patient demographics.

**Table 1:** Variable summary by treatment group. ^c^Chi-squared test, ^t^T-test,^ f^Fisher's exact test, ^T^Tobit regression. S/P = serum/plasma; SD = standard deviation; IQR = interquartile range; eGFR = estimated glomerular filtration rate; preop = preoperative; postop = postoperative. <7 = number of left-censored TP Urine values in each cohort. Missing values by group: Preop Cystatin C = 0/5, Postop Creatinine (S/P) Year 1 = 2/2, Postop eGFR Year 1 = 2/3, Preop TP Urine = 0/2, Preop TP Urine 24 hrs = 12/11, TP Urine Year 1 = 2/3.

Variable	ERAS (N = 38)	Placebo (N = 34)	p value
Age			
Mean (SD)	43.7 (10.5)	44.0 (12.7)	0.91^t^
Median (IQR)	42.5 (34.2, 50.8)	43.0 (35.0, 53.0)	-
Range	(25.0, 66.0)	(23.0, 69.0)	-
Height (cm)			
Mean (SD)	169.0 (9.8)	171.2 (9.2)	0.33^t^
Median (IQR)	170.2 (162.0, 172.7)	170.2 (166.4, 179.0)	-
Range	(149.0, 195.6)	(152.4, 190.5)	-
Weight (kg)			
Mean (SD)	75.8 (16.2)	80.0 (14.4)	0.24^t^
Median (IQR)	76.4 (62.8, 86.4)	79.0 (70.7, 89.3)	-
Range	(50.9, 123.0)	(56.2, 117.0)	-
BMI (kg/m^2^)			
Mean (SD)	26.2 (4.5)	27.2 (3.5)	0.32^t^
Median (IQR)	26.6 (22.2, 29.6)	27.5 (24.2, 28.9)	-
Range	(17.1, 34.8)	(21.0, 36.0)	-
Sex			
Female	30 (78.9%)	18 (52.9%)	0.019^c^
Male	8 (21.1%)	16 (47.1%)	-
Race			
American Indian and Alaska Native	1 (2.6%)	0 (0%)	0.47^f^
Black or African American	1 (2.6%)	0 (0%)	-
Other	1 (2.6%)	3 (8.8%)	-
Refused	1 (2.6%)	0 (0%)	-
White-Caucasian	34 (89.5%)	31 (91.2%)	-
Ethnicity			
Hispanic/Latino	3 (7.9%)	2 (5.9%)	1.00^f^
Not Hispanic/Latino	33 (86.8%)	32 (94.1%)	-
Opts Out	1 (2.6%)	0 (0%)	-
Unknown/Not Abvai	1 (2.6%)	0 (0%)	-
Preop Creatinine, S/P (mg/dL)			
Mean (SD)	0.8 (0.2)	0.9 (0.1)	0.16^t^
Median (IQR)	0.8 (0.7, 0.9)	0.9 (0.8, 1.0)	-
Range	(0.6, 1.2)	(0.6, 1.2)	-
Preop TP, S/P (g/dL)			
Mean (SD)	7.4 (0.5)	7.4 (0.4)	1.00^t^
Median (IQR)	7.4 (7.2, 7.6)	7.5 (7.1, 7.6)	-
Range	(6.2, 8.3)	(6.3, 7.9)	-
Preop Cystatin C			
Mean (SD)	0.7 (0.1)	0.7 (0.1)	0.50^t^
Median (IQR)	0.7 (0.6, 0.8)	0.6 (0.6, 0.7)	-
Range	(0.5, 1.0)	(0.5, 0.8)	-
Preop eGFR (mL/min/1.73 m^2^)			
Mean (SD)	93.1 (14.4)	93.1 (14.0)	1.00^t^
Median (IQR)	93.0 (82.8, 103.0)	95.0 (82.5, 104.8)	-
Range	(64.0, 123.0)	(68.0, 118.0)	-
Postop Creatinine, S/P Day 1 (mg/dL)			
Mean (SD)	1.3 (0.3)	1.3 (0.2)	0.49^t^
Median (IQR)	1.2 (1.1, 1.4)	1.3 (1.2, 1.5)	-
Range	(0.7, 1.8)	(0.9, 1.9)	-
Postop Creatinine, S/P Year 1 (mg/dL)			
Mean (SD)	1.1 (0.2)	1.2 (0.2)	0.09^t^
Median (IQR)	1.1 (1.0, 1.2)	1.2 (1.0, 1.4)	-
Range	(0.6, 1.7)	(0.8, 1.6)	-
Postop eGFR 1 Day (mL/min/1.73 m^2^)			
Mean (SD)	56.1 (12.2)	57.1 (9.7)	0.68^t^
Median (IQR)	54.0 (49.0, 58.8)	56.0 (52.0, 60.5)	-
Range	(36.0, 97.0)	(41.0, 82.0)	-
Postop eGFR 1 yr (mL/min/1.73 m^2^)			
Mean (SD)	67.6 (16.9)	64.2 (11.9)	0.33^t^
Median (IQR)	61.5 (56.8, 72.5)	61.5 (55.8, 71.0)	-
Range	(40.0, 114.0)	(42.0, 100.0)	-
TP Urine pre, ≥7 (mg/dL)			
Mean (SD)	14.5 (17.8)	10.1 (3.8)	0.4^T^
Median (IQR)	9.9 (8.2, 11.8)	9.4 (8.2, 10.0)	
Range	(7.0, 76.0)	(7.0, 20.0)	
<7:	24 (63.2%)	22 (68.8%)	
TP Urine 24 hrs pre* (mg/dL)			
Mean (SD)	137.6 (58.1)	160.7 (84.4)	0.28^t^
Median (IQR)	137.9 (119.2, 160.2)	159.0 (92.5, 192.0)	
Range	(0.1, 317.9)	(45.0, 419.9)	
TP Urine Post 1 yr > 7 (mg/dL)			
Mean (SD)	10.4 (3.4)	14.9 (5.8)	
Median (IQR)	9.0 (7.5, 12.5)	12.0 (11.0, 19.0)	0.025^T^
Range	(7.0, 17.0)	(8.0, 26.5)	
<7:	21 (58.3%)	14 (45.2%)	

There was no significant difference in postop eGFR at one year between ERAS and placebo adjusting for preop eGFR (3.07 ml/min/1.73 m2, 95% CI: -2.14, 8.28; p = 0.24). However, preop eGFR was significantly associated with postop eGFR, where on average a 1-unit (ml/min/1.73 m2) increase in preop eGFR was associated with a 0.74-unit higher postop eGFR (0.74 ml/min/1.73 m2, 95% CI: 0.55, 0.93; p < 0.001) (Table 2).

**Table 2:** Comparing postop eGFR (ml/min/m2) at one year between treatment groups adjusting for preop eGFR. CI = confidence interval; ERAS = enhanced recovery after surgery; eGFR = estimated glomerular filtration rate.

Variable	Coefficient (95% CI)	p value
Cohort ERAS	3.07 (-2.14, 8.28)	0.24
Preop eGFR	0.74 (0.55, 0.93)	<0.001

TP urine at one-year postoperative was significantly lower in the ERAS cohort by 4.7 mg/dL on average (95% CI 0.48 ~ 8.82, p = 0.025) as illustrated in Table 3.

**Table 3:** Comparing postop TP urine (mg/dL) at one year between treatment groups. ERAS = enhanced recovery after surgery.

Variable	Coefficient (95% CI)	p value
Cohort ERAS vs. placebo	-4.70 (-8.82, -0.58)	0.025

## Discussion

Narcotic pain medications after LDKT can delay a donor's return to normal daily function. The constant exposure to opioid-mediated pain analgesia can potentially lead to opioid-use disorder, which currently is an epidemic in the United States [15-17]. Additional costs can be incurred by the health-care system, donors from lost wages, or even act as a potential deterrent for live organ donation candidates [18]. The Centers for Disease Control and Prevention estimates 72 billion dollars each year in medical costs are a consequence of prescription opioid misuse with nearly 19,000 overdose-related deaths occurring in the United States annually [19]. While prescription narcotic use among the LDKT population is not well described in the literature [18], it is an acknowledged point among the medical community that inpatient narcotic administration predisposes patients for potential long-term narcotic misuse [17]. The ERAS protocol using ketorolac did reduce the need for narcotic use after surgery [20].

The primary outcome of the two original trials was safety measure by preservation of renal function. This post hoc analysis did not show a difference in renal function (eGFR) between the two groups at one year after donation. However, at one year, the ERAS group had significantly less TP in the urine than the placebo group. Thus, perioperative exposure to ketorolac did not negatively affect renal function and surprisingly may be protective as evidenced by the significantly less proteinuria one year after donation. Differences in the urine TP between the two groups are of unknown significance and cannot be conclusive of protection to kidney function with ketorolac use.

A hypothesis of why TP was lower in the ERAS group was less exposure to dehydration. Meaning because the ERAS group perioperatively has a significantly shorter length of stay and required less narcotics; this may have allowed the patients to return to normal activities of daily life including developing a normalized bowel function and diet. Thus, after surgery the donor had less chance of exposure to dehydration. We were not able to determine why the TP was lower because the urine protein measure is of uncertain clinical significance. More clinically relevant measures of urinary protein are 24-hour timed collection or protein-to-creatinine ratio. While albuminuria (timed albumin excretion rate or albumin-to-creatinine ratio) is a better measure of renal damage according to the 2012 KDIGO CKD guidelines, proteinuria was the only consistent data available [21]. We focused on renal function as the primary endpoint; but, a larger multicenter study could expand to focus on longer follow-up after discharge examining quality of life and narcotic use [22].

The limitations of this study are that it was from a single center. The sample size is very small, which impacts statistical power. While the study was randomized, there remains a significant gender difference between the experimental and placebo arms, which has potential for bias. The ERAS group has 21% men, and the placebo group has 47% men. We did use the CKD-EPI formula that incorporates gender to offset this potential issue. The study comprised of 90%-91% Caucasian patients in both arms. The lack of racial diversity limits the generalizability of the results.

Ketorolac was administered before the patient was awakened from surgery and continued for 48 hours after surgery or less if discharge was earlier. Ketorolac is indicated for the short-term (up to five days in adults) management of moderately severe acute pain. Many transplant centers use ketorolac for non-opioid pain management in donors [23-25]; however, before these studies, there was no level 1 data to support its widespread usage. This study does contradict Takahashi et al. who found lower GFR and urinary alb/Cr ratio at one year following ketorolac use [22].

## Conclusions

This study was conceived and designed to answer the question of whether short-term perioperative exposure of kidney donors to ketorolac is safe. A one-year post hoc analysis of an ERAS pathway following live donor kidney surgery demonstrates that estimated GFR is not significantly affected postoperatively when compared to standard of care plus placebo in live kidney donor patients. The ERAS pathway also reduces the risk of opioid-related substance use disorder as well as reduces the cost of care for live donor patients. These results support the use of ketorolac in ERAS protocols for live kidney donation.
